# MEIS1-mediated Apoptosis via TNFR1 in Endometriosis

**DOI:** 10.1007/s43032-025-01801-1

**Published:** 2025-02-11

**Authors:** Wenwen Wang, Fangfang Fu, Yan Li, Sha Li, Ming Yuan, Tian Wang, Wu Ren, Jia Wei, Dan Chen, Shixuan Wang, Xiangyi Ma, Zhangying Wu

**Affiliations:** 1 Department of Obstetrics and Gynecology, Tongji Hospital, Tongji Medical College, Huazhong University of Science and Technology, 1095 Jiefang Anv., Wuhan, Hubei 430030 P.R. China; 2 Department of Obstetrics and Gynecology, The Affiliated Hospital of Guizhou Medical University, Guiyang, Guizhou 550000 P.R. China; 3 Department of Obstetrics and Gynecology, The Central Hospital of Wuhan, Tongji Medical College, Huazhong University of Science and Technology, Wuhan, Hubei 430014 P.R. China

**Keywords:** Endometriosis, MEIS1, TNFR1, Apoptosis, Caspase pathway, Proliferation

## Abstract

Abnormal apoptosis both maintains endometrial cell growth and induces endometrial pathogenesis. The etiology of endometriosis is unclear and no treatment is curative. Therefore, the aim herein was to identify genes involved in the pathogenesis of endometriosis. Using the data from our previous results and RNA sequencing data of normal endometrial tissue and ovarian endometrioma (OMA) tissue, along with Gene Expression Omnibus (GEO) dataset on endometriosis, we identified an apoptotic-related gene, meis homeobox I (MEIS1). Normal endometrium, eutopic endometrium and ectopic endometriotic tissues were used to detect MEIS1. Primary normal endometrial and eutopic endometrial stromal cells were isolated and cultured for exploring the function of MEIS1 and related pathways. A mouse endometriosis model was used to verify the therapeutic effects of MEIS1. The mRNA and protein of MEIS1 in tissues from patients with endometriosis were decreased. Overexpression of MEIS1 induced the apoptosis of primary eutopic endometrium stromal cells by regulating TNFR1. Using Cell Counting Kit 8 (CCK8) assay and EdU assay, we found that knockdown of MEIS1 promoted the proliferation of primary normal endometrium stromal cells. We also observe that upregulated MEIS1 may lead to caspase pathway activation, promoting endometrial cell apoptosis. Furthermore, MEIS1 lentivirus inhibited endometriotic lesion formation and induced apoptosis in the mouse endometriosis model. These cumulative findings suggest that MEIS1 may mediate apoptosis by initiating TNFR1 in endometrial cells via the caspase pathway.

**Supplementary Information:**

The online version contains supplementary material available at 10.1007/s43032-025-01801-1.

## Introduction

Endometriosis is an estrogen-dependent, benign gynecological disease, defined as the appearance of endometrial tissue outside the uterine cavity. An estimated 6–10% of women of childbearing age suffer from endometriosis [1], the main symptoms of which include pelvic pain, dysmenorrhea, and infertility, potentially causing poor quality of life and heavy economic burden among 35–50% of reproductive-age patients [2]. The common phenotypes are superficial (or peritoneal) endometriosis, ovarian endometrioma (OMA), and deep infiltrating endometriosis, with ~ 44% of patients suffering from OMA [3]. The pathogenesis of endometriosis is currently unclear, and the widely endorsed retrograde menstruation theory cannot explain the mechanisms of all phenotypes. However, dysregulation and resistance of apoptosis allows ectopic endometrial tissue to evade immune clearance [4], a process in which various apoptotic pathways may play crucial roles [5, 6].

Apoptosis activation can be triggered by different stimuli, including physiological and pathological factors.

Apoptosis-promoting factors in endometriosis include progestogens, Fas and Fas ligands (Fas/FasL), DNA fragmentation factor, and P53 [7–9]. Apoptosis inhibitors include estradiol, nuclear factor κB (NF-κB), serine-threonine kinase (AKT), B-cell lymphoma-2 (BCL-2), and survivin. Abnormal regulation of the Fas/FasL system prevented ectopic endometrial cells from thriving in ectopic plantings through the Fas/FasL-mediated apoptosis pathway [10, 11]. Other researchers have found that NF-κB could upregulate BCL-2 expression by activating the AKT/P13K pathway, which could also increase expression of the X-linked inhibitor of apoptosis protein, and ultimately reduce apoptosis in patients with endometriosis [12].

In the previous study, we conducted a pooling-based genome-wide association study (GWAS) to identify OMA susceptibility loci in Chinese Han women from central China [13]. The top three loci were: insulin-like growth factor I receptor; chromosome 7 open reading frame 50; and meis homeobox I (MEIS1). We then used RNA sequencing, combined with endometriosis database (GSE7305) [14], to analyze transcriptional profiles to characterize OMA. MEIS1, a homeobox gene, presented a high relation to OMA.

MEIS1 belongs to the homologous cassette protein TALE family, and is an important transcription factor in cell proliferation, apoptosis, and differentiation [15, 16]. A conserved N-terminal bidirectional MEINOX domain can mediate protein–protein interactions [17]. In 1995, it was first identified as a viral integration site in the BXH-2 mouse model of leukemia [18]. Subsequently, a great deal of research has been focused on the processes of proliferation, apoptosis, and differentiation of MEIS1 in leukemia [19–22], solid tumors [23], and limb development [24]. Taylor et al. firstly reported MEIS1 was expressed in human endometrium stroma throughout the menstrual cycle [25]. Unexpectedly, Qian et al. found downregulation of MEIS1 expression in eutopic endometrium compared with normal endometrium, potentially contributing to implantation failure in endometriosis [26]. Though MEIS1 may be a mediator between sex steroids and key genes for uterine receptivity, its role in the pathogenesis of endometriosis has remained uncertain.

Herein, we demonstrated that MEIS1 level was decreased in eutopic and endometriotic cells of endometriosis compared with normal endometrium. A higher MEIS1 level induced apoptosis of endometrial stromal cells in endometriosis, in which MEIS1 activated TNFR1 transcription activity to induce the apoptosis caspase pathway. Furthermore, improved MEIS1 signaling prevented mouse endometriotic lesion formation. These results suggested the potential for developing MEIS1 as a targeted endometriosis therapy.

## Materials and Methods

### Ethical Statement

This study was approved by the Ethical Committee of Tongji Medical College, Huazhong University of Science and Technology (approval number: TJ-IRB2011S462). Animal experiments were approved by the Laboratory Animal Welfare & Ethics Committee of TongJi Hospital (ethical approval protocol number: TJH-202101010).

### Clinical Participants and Sample Collection

22 patients with ovarian endometriosis and 22 without endometriosis participated from August 2011 to April 2021 at the Department of Obstetrics and Gynecology in Tongji Hospital, Tongji Medical College, Huazhong University of Science and Technology. Basic information about the participants was listed in Table 1. The OMA tissues and eutopic endometrium (Eut-E) tissues were collected from participants with ovarian endometriosis, and the normal endometrium (Nor-E) tissues were obtained from participants without ovarian endometriosis. No participant received any hormonal therapy for three months prior to surgery, was pregnant or lactating, had any genital tract anomaly or malignancy, or had a serious chronic disease.

**Table 1:** Characteristics of patients with and without endometriosis

Characteristic	OMA/Eut-E	Nor-E
Number of patients	22	22
Age (years)	32 ± 7.68	41.18 ± 7.12
Menstrual Cycle		
Proliferative Phase	12	12
Secretory Phase	10	10
rAFS Classification		
III	19	
IV	3	

OMA: Ovarian endometrioma; Eut-E: Eutopic endometrium; Nor-E: Normal endometrium; rAFS: revised American fertility society classification of endometriosis

After surgical removal, tissues were divided into three parts. One part was conserved in Dulbecco’s Modified Eagle’s Medium Nutrient Mixture F-12 HAM (DMEM/F12) with 10% fetal bovine serum (FBS) medium and kept on ice until stromal cell isolation. The second part was snap frozen in liquid N_2_ and stored at − 80 °C. The last part was fixed with 4% paraformaldehyde.

### RNA Sequencing and Analysis

Total RNA was extracted from five Nor-E tissue samples and five OMA tissue samples using TRIzol (Invitrogen, Carlsbad, CA, USA) according to the manufacturer’s instructions. Subsequently, total RNA was qualified and quantified using kaiaoK5500^®^ Spectrophotometer (Kaiao, Beijing, China) and the RNA Nano 6000 Assay Kit from the Bioanalyzer 2100 system (Agilent Technologies, CA, USA). A total of 2 µg RNA per sample was used as the input material for RNA sample preparations. Sequencing libraries were generated using the NEBNext^®^ Ultra™ RNA Library Prep Kit for Illumina^®^ (#E7530L, NEB, Ipswich, MA, USA) following the manufacturer’s recommendations, and index codes were added to attribute sequences for each sample. mRNA sequencing analysis was performed using Annoroad Gene Technology. To identify novel genes associated with endometriosis, a heatmap (*P* < 0.05, fold change ≥1.5) was constructed for our RNA sequence, including normal and endometriotic specimens.

### Primary Endometrial Stromal Cell Culture

Human Nor-E tissues and Eut-E tissues were washed with phosphate-buffered saline (PBS) and finely minced. Then, type IV collagenase (1 mg/mL) and DNase I (100 µg/mL) were used to digest tissues in DMEM/F-12 for 60 min at 37℃. Stromal cells were filtrated through a 40 μm strainer to separate the normal endometrial stromal cells (NESCs) and eutopic endometrial stromal cells (EESCs), and then plated on a 75 cm^2^ tissue culture Falcon flask (BD Biosciences, San Jose, CA, USA) in DMEM/F12 medium with 10% FBS and 1% Penicillin-streptomycin in an incubator with 5% CO_2_ at 37 °C. The stromal cells were identified by cell immunochemistry of vimentin (Supplementary Fig. 1).

### RNA Isolation and Quantitative Real-time Polymerase Chain Reaction

Total RNA was extracted from tissues or cultured cells using TRIzol reagent (Thermo Fisher Scientific, Waltham, MA, USA) and concentrations of RNA were quantified by the Nanodrop 2000 (Thermo Fisher Scientific). Reverse transcription was conducted using the ReverTra Ace qPCR RT kit (Toyobo, Japan) and real-time polymerase chain reaction (PCR) was performed using the SYBR Green qPCR Master Mix (DBI, USA) on a CFX Connect real-time system (Bio-Rad, USA) according to the manufacturer’s protocols. Primer sequences were listed in Table 2. This part of the experiment was repeated at least three times. The 2^−ΔΔCT^ method was used to evaluate the expression of MEIS1 using 18 S rRNA or β-actin as references.

**Table 2:** Primer sequences

Gene Symbol	Forward	Reverse
MEIS1	AACAGCAGTGAGCAAGGTGATG	GAACAGCCACGCCCTCAT
18 S rRNA	GTAACCCGTTGAACCCCATT	CCATCCAATCGGTAGTAGCG
β-actin	CATGTACGTTGCTATCCAGGC	CTCCTTAATGTCACGCACGAT

### Western Blotting

Total protein was extracted from tissues and cultured cells using RIPA lysis buffer-containing protease inhibitor cocktail (Roche, USA). The concentration was quantified using Nanodrop 2000 (Thermo Fisher Scientific). Protein was resolved by SDS-PAGE gels and transferred to PVDF membranes using an electroblotter (Bio-Rad, USA), which was blocked with 5.0% nonfat milk and incubated with primary antibodies at 4 °C overnight. After washing with PBS, the membrane was incubated with a second antibody for 1 h, then washed with PBS. ECL (NEL104/105, PerkinElmer™ Life Sciences, Inc., Boston, MA, USA) was used for immunodetection. Images were quantified using ImageJ image analysis software (Wayne Rasband, NIH, USA). The antibodies used were as follows: MEIS1 (1:1000, Abcam, ab229962), TNFR1 (1:500, Proteintech, 21574-1-AP), caspase8 (1:500, Immunoway, YT6191), cleaved-caspase8 (1:500, Immunoway, YC0011), IGF-II (1:500, ABclonal, A2086), and β-actin (1:500, ABclonal, AC038). This part of the experiment was repeated at least three times.

### Cell Transfection, siRNA and Lentivirus

Primary cultured endometrial stromal cells from patients with and without endometriosis were planted in 6-well or 96-well plates. When it reached 70% confluence, the culture medium was changed to serum-free for 24 hours. To silence MEIS1 expression, stromal cells from Nor-E tissues were transfected with MEIS1 siRNA or control siRNA (RiboBio, Guangzhou, China) using Lipofectamine 2000 (Invitrogen, CA, USA) at a final concentration of 50 nM for subsequent proliferation experiments. Upregulation of MEIS1 was conducted in stromal cells from Eut-E tissues by using MEIS1 lentivirus (Genechem, Shanghai, China) with 1 × 10^6^TU/ml for subsequent apoptosis experiments. The targeted sequence by siRNA of MEIS1 followed as below: 5’ -GATTCAAGCCATACAAGTA-3’. The cDNA sequence of MEIS1 was referred to NM_002398, and cloned into the GV492 vector.

### Human Apoptosis Antibody Array

Proteins from 1 × 10^^7^ EESCs with and without MEIS1 overexpression were extracted using 1X Cell Lysis Buffer. Lysate was incubated overnight with RayBio Human Apoptosis Antibody (RayBio Human Apoptosis Antibody Array, Ca# AAH-APO-G1-4, GA, USA). Antibody array slides were then washed, and a biotinylated antibody mixture was used to detect apoptosis-related proteins. After incubation with fluorescence dye, signals were scanned with a fluorescence scanner. The antibody array map was present in Table 3.

**Table 3:** Human apoptosis antibody map

Pos	Pos	Neg	Neg	Blank	Blank	bad	bax	bcl-2	bcl-w	BID	BIM	Caspase3	caspase8
Pos	Pos	Neg	Neg	Blank	Blank	bad	bax	bcl-2	bcl-w	BID	BIM	Caspase3	caspase8
CD40	CD40L	cIAP-2	cytoC	DR6	Fas	FasL	GAPDH	HSP27	HSP60	HSP70	HTRA	IGF-I	IGF-II
CD40	CD40L	cIAP-2	cytoC	DR6	Fas	FasL	GAPDH	HSP27	HSP60	HSP70	HTRA	IGF-I	IGF-II
IGFBP-1	IGFBP-2	IGFBP-3	IGFBP-4	IGFBP-5	IGFBP-6	IGF-1sR	livin	p21	p27	p53	SMAC	Survivin	sTNF-R1
IGFBP-1	IGFBP-2	IGFBP-3	IGFBP-4	IGFBP-5	IGFBP-6	IGF-1sR	livin	p21	p27	p53	SMAC	Survivin	sTNF-R1
sTNF-R2	TNF-alpha	TNF-beta	TRAILR-1	TRAILR-2	TRAILR-3	TRAILR-4	XIAP	Blank	Blank	Neg	Neg	Neg	Pos
sTNF-R2	TNF-alpha	TNF-beta	TRAILR-1	TRAILR-2	TRAILR-3	TRAILR-4	XIAP	Blank	Blank	Neg	Neg	Neg	Pos

### Flow Cytometry

Treated stromal cells were washed with PBS and suspended with a binding buffer. Cells were stained with Annexin V-fluorescein isothiocyanate (FITC) and propidium iodide, and incubated at room temperature for 10 min by using the FITC Annexin Apoptosis Detection Kit (BD Biosciences, USA, Catalog number:556547). Stained cells were quantified by flow cytometry and the data were analyzed using FlowJo 10.8 (BD Biosciences, USA). This part of the experiment was repeated at least three times.

### Cell Proliferation Assay

Cells were seeded into 96-well plates at a density of 5000 cells per well in triplicate, and assessed every 24 h for 3 days using the Cell Counting Kit 8 (Dojindo Laboratories, Kumamoto, Japan, CK04-500T) according to the manufacturer’s instructions. This part of the experiment was repeated at least three times. The absorbance of 450 nm was used to assure the living cells.

The proliferation rate of NESCs was detected by using the Click-iT EdU cell proliferation Kit (iFluor 555) (Servicebio, G1602) according to the manufacturer’s instructions. In brief, the treated NESCs were grown in 96-well plates. After 24 h, the cells were incubated in complete DMEM/F-12 with 10µM EdU for 3 h, then followed by fixation, permeabilization and EdU click reaction. After removing the reaction solution, 4′,6-diamidino-2-phenylindole (DAPI) was used to stain the nucleus. Images were captured with fluorescence microscopy (Olympus, Japan).

### Immunohistochemistry

Tissue specimens were formalin-fixed and paraffin-embedded. Then, 4 μm sections were cut, deparaffinized, and blocked by methanol and 0.3% H_2_O_2_. After incubating with primary antibody overnight: MEIS1 (1:1000, Abcam, ab229962), TNFR1(1:50, Proteintech, 21574-1-AP), enzyme-conjugated secondary antibodies were applied, and the staining was visualized using 3,3′-diaminobenzidine tetrahydrochloride (DAB, Dako, Denmark) and hematoxylin. Expression levels of MEISI and TNFR1 proteins were evaluated based on the mean density using Image Pro-Plus software 6.0 (IPP 6.0).

### Construction of TNFR1 Promoter Vector and Dual-luciferase Reporter Assay

We synthesized the 988 bp bases upstream of the transcription start site of *Tnfr1* gene, constructed this sequence into the PGL3 basic vector, and named it as TNFR1-pGL3b. The PGL3 basic vector was used as a negative control.

For the promoter luciferase reporter assay, HEK293T cells were inoculated in 24-well plates. When 70% cell fusion was reached, the cells were transfected with 450 ng of the PGL3 basic vector, TNFR1-pGL3b plasmids, TNFR1-pGL3b plasmids plus 1 × 10^6TU/ml MEIS1 lentivirus and 50 ng of pRL-CMV Renilla luciferase expression vector, which was used to normalize the transfection efficiencies. After 48 h, the luciferase activity was measured using the dual-luciferase reporter assay system (Promega, E1910) according to the manufacturer’s instructions. This part of the experiment was repeated at least three times.

### Animal Experiment

12 seven-week-old female BALB/c-nu were obtained from Beijing Vital River Laboratory Animal Technology Co., Ltd. To set up a xenograft model, endometrial tissues were obtained from patients with endometriosis, divided into 2 mm × 2 mm pieces and sutured to the peritoneal wall. Four mice died during a four-week observation period after surgery. The remaining eight mice were randomly assigned to the LV-MEIS1 group and the LV-CON group. Then, every three days the mice were administered intraperitoneal 10^^7^ TU lentivirus-MEIS1 (named as LV-MEIS1 group) or lentivirus-control (named as LV-CON group). After intraperitoneal injection for 15 days, the ectopic lesion was incised, separated, weighted, and embedded in paraffin.

### Terminal-deoxynucleotidyl Transferase-mediated Nick End Labeling Staining

Sections were deparaffinized, incubated with proteinase K (2 mg/mL), and permeabilized with 0.2% Triton X-100 in PBS. Then, endogenous peroxidase activity was blocked by 3% H_2_O_2_, terminal-deoxynucleotidyl transferase-mediated nick end labeling (TUNEL) assay kit (Roche, IN, USA) was used according to the manufacturer’s instructions, and the nuclei were counterstained with DAPI. Images were captured with fluorescence microscopy (Olympus, Japan).

### Statistical Analysis

The results are presented as mean ± standard deviation. Between-groups differences were analyzed using Student’s *t* test or one-way analysis of variance. Analysis of characteristics between endometriosis patients and controls were assessed using Mann-Whitney U test. Graphs were made using a commercial statistical software (GraphPad Prism 5.02, GraphPad Software, San Diego, CA). SPSS 23.0 (IBM Corp, Armonk, NY, USA) was used to perform statistical analyses. Results were the means of three independent experiments. *P* < 0.05 was considered statistically significant. ns: not significant, *p < 0.05, **p < 0.01, and ***p < 0.001.

## Results

### MEIS1 Expression was Decreased in Eutopic Endometrium and OMA Tissues of Patients with Endometriosis

**Fig. 1:**
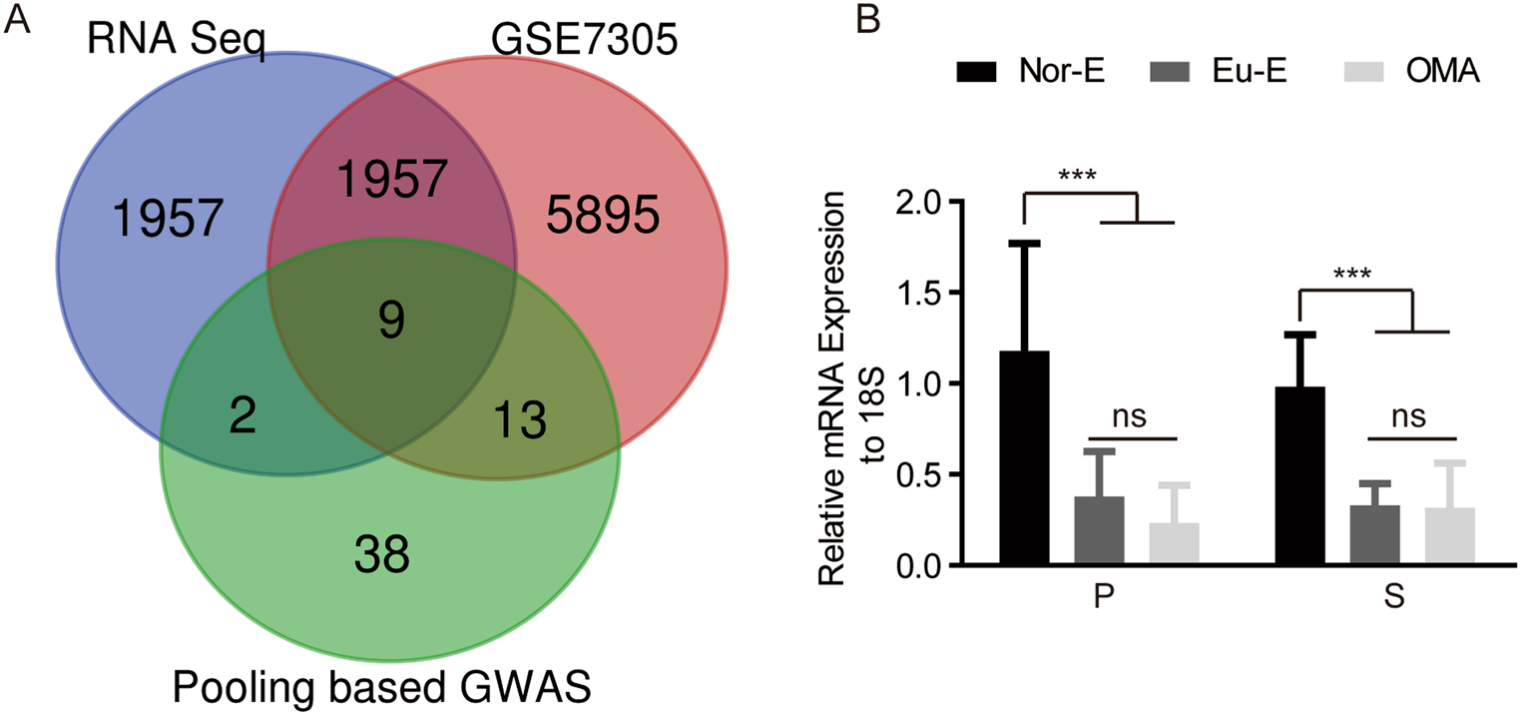
MEIS1 was decreased in endometriotic tissues. (**A**) Venn diagram showing the overlapping genes identified by RNA-seq, endometriosis database (GSE7305) and pooling based GWAS. These genes are NEK2, DNM1, MEIS1, ESR1, DACH, SCN5A, IL7R, UBE2T and ESR2. (**B**) The mRNA expression of MEIS1 in normal endometrium, ectopic endometrium tissues and endometriotic tissues from different menstrual cycle. ***p < 0.001. Nor-E: normal endometrium, Eut-E: eutopic endometrium, OMA: ovarian endometrioma, P: proliferative phase, S: secretory phase

To identify OMA susceptibility genes in Chinese Han women, we analyzed genes in our pooling-based GWAS [13], RNA sequencing (Supplementary Fig. 2), and public data set GSE7305 using normal endometrial and endometriotic tissues [14]. MEIS1, identified as the gene of interest, is an important transcription factor in cell proliferation and apoptosis (Fig. 1A). mRNA expression of MEIS1 was lower in endometriotic tissues compared with normal endometrium in different menstrual cycle stages, but did not differ between eutopic endometrium and ectopic endometriotic tissue, which was independent of menstrual cycle (Fig. 1B).

**Fig. 2:**
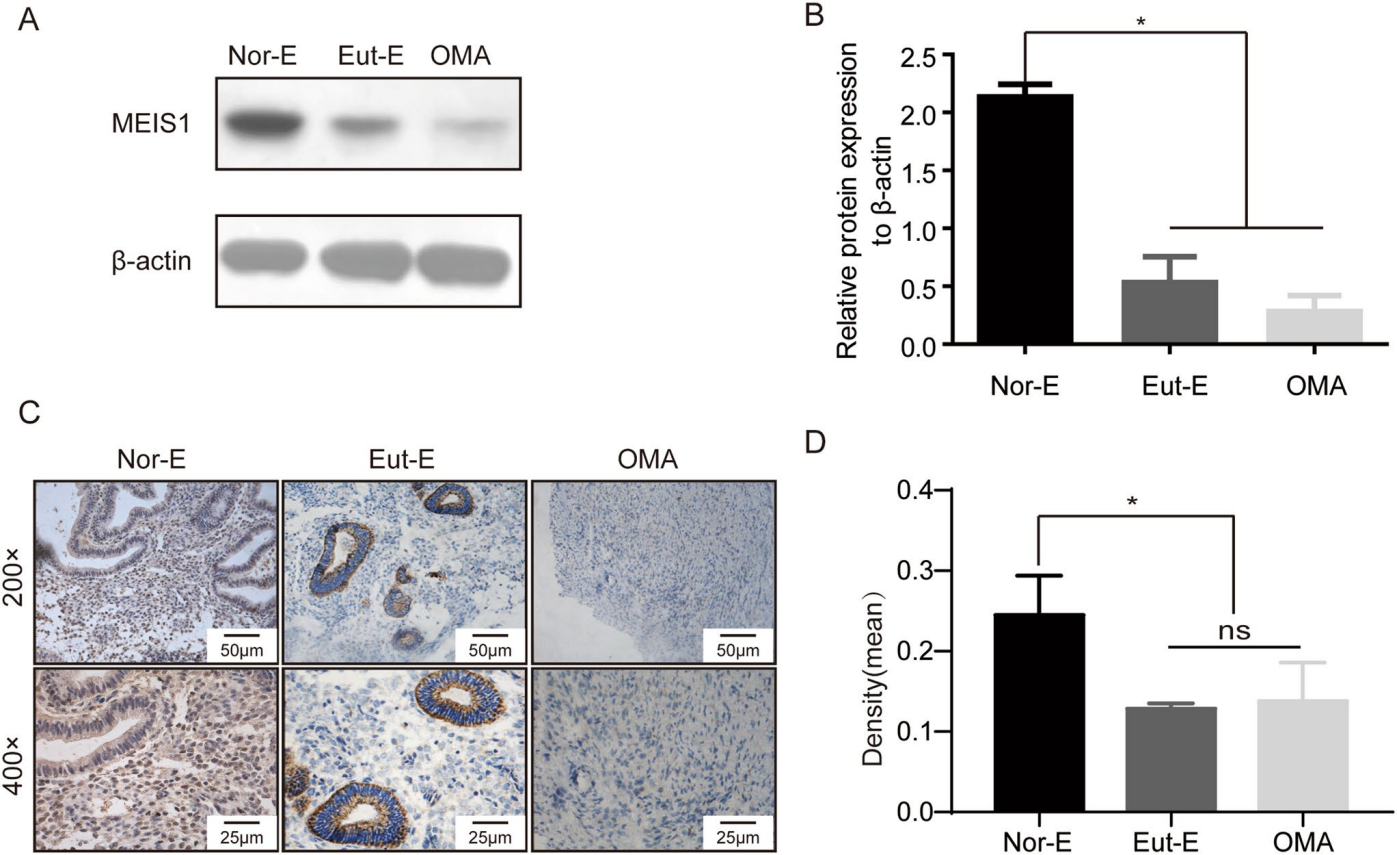
The protein expression of MEIS1 was upregulated in stromal cells from normal endometrium. (**A**) Western blotting of MEIS1 protein level in normal endometrium, ectopic endometrium tissues and ovarian endometrioma tissues. (**B**) The quantitative result of A from three independent experiments. Date is presented as mean ± SD. *p < 0.01. (**C**) The representative pictures of MEIS1 in normal endometrium, ectopic endometrium tissues and ovarian endometrioma tissues using IHC assay. (**D**) The quantitative result of C from 22 normal endometrium, 22 ectopic endometrium tissues and 22 ovarian endometrioma tissues by using Image Pro-Plus (IPP). Date is presented as mean ± SD.*p < 0.01. Nor-E: normal endometrium, Eut-E: eutopic endometrium, OMA: ovarian endometrioma

Next, MEIS1 protein expression was detected in women with and without endometriosis using Western blotting and immnohistochemistry(IHC). As shown in Fig. 2, MEIS1 was overexpressed in cell nucleus and cytoplasm of both epithelial and stromal cells from normal endometrium compared with eutopic endometrium and ectopic endometriotic tissues of endometriosis, especially in stromal cells. These results suggested that abnormal MEIS1 expression might be associated with the pathogenesis of endometriosis.

### Abnormal MEIS1 Expression in Normal and Eutopic Endometrium Affected Stromal Cell Proliferation and Apoptosis

**Fig. 3:**
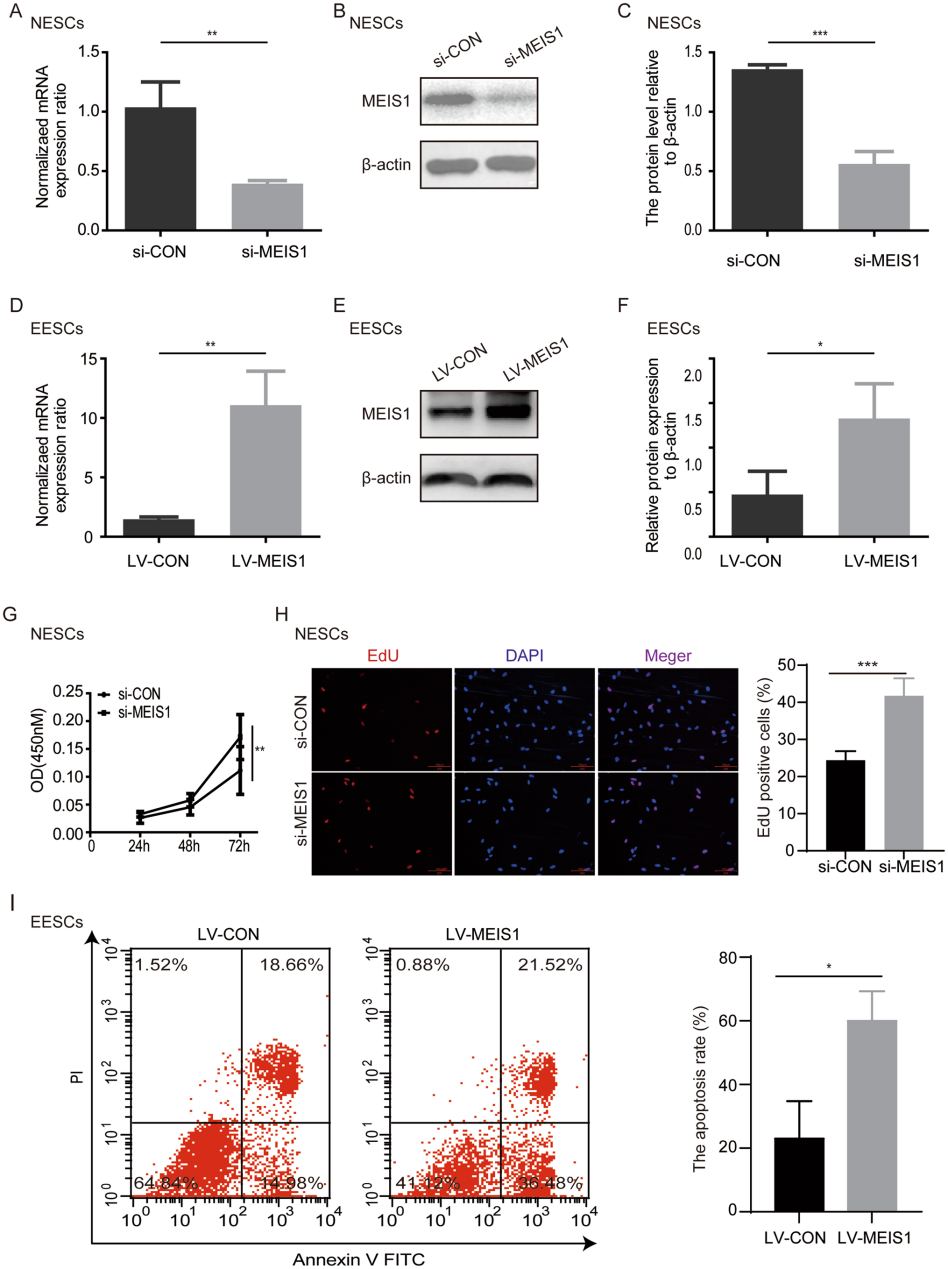
The role of MEIS1 in stromal cells of endometrium. (**A**) The mRNA expression of MEIS1 was detected by real-time PRC in NESCs treated with MEIS1 siRNA from three independent experiments. β-actin was used as the control gene. Date is presented as mean ± SD. **p < 0.01. (**B**) The proteins expression of MEIS1 was detected by Western blotting in NESCs treated with MEIS1 or control siRNA. β-actin was used as the control gene. (**C**) The relative protein level of MEIS1 to β-actin in (B). Date is presented as mean ± SD. ***p < 0.001. (**D**) The mRNA expression of MEIS1 was detected by real-time PRC in EESCs treated with MEIS1 lentivirus from three independent experiments. β-actin was used as the control gene. Date is presented as mean ± SD. **p < 0.01. (**E**) The proteins expression of MEIS1 was detected by Western blotting in EESCs after treated with MEIS1 or control lentivirus. β-actin was used as the control gene. (**F**) The relative protein level of MEIS1 to β-actin in (**E**). Date is presented as mean ± SD. ***p < 0.001. (**G**) The proliferation activity of NESCs after silencing MEIS1 through siRNA assessed by CCK8 assay. **p < 0.01. (**H**) The proliferation of NESCs after silencing MEIS1 through siRNA was detected by EdU assay. ***p < 0.001. (**I**) The apoptosis rate of EESCs after upregulating MEIS1 via lentivirus detected by flow cytometry. *p < 0.05. NESCs: Normal endometrial stromal cells, EESCs: eutopic endometrial stromal cells

To investigate the biological function of MEIS1 on endometriosis pathogenesis, we downregulated MEIS1 expression by siRNA in NESCs (Fig. 3A, B and C). Further, MEIS1 expression was upregulated in EESCs (Fig. 3D, E and F). Cell proliferation assay, conducted by using the CCK8 assay and EdU assay, revealed that downregulation of MEIS1 increased proliferation of endometrial stromal cells (Fig. 3G and H). Flow cytometry analysis revealed that the apoptosis rate of EESCs significantly increased after transfecting LV-MEIS1 (Fig. 3I). These results indicated that downregulation of MEIS1 in eutopic stromal cells of endometriosis might promote cell proliferation and restrain cell apoptosis, which might contribute to endometriosis progression.

### MEIS1 Promoted Apoptosis in Stromal Cells by Initiating the TNFR1-mediated Caspase Pathway

**Fig. 4:**
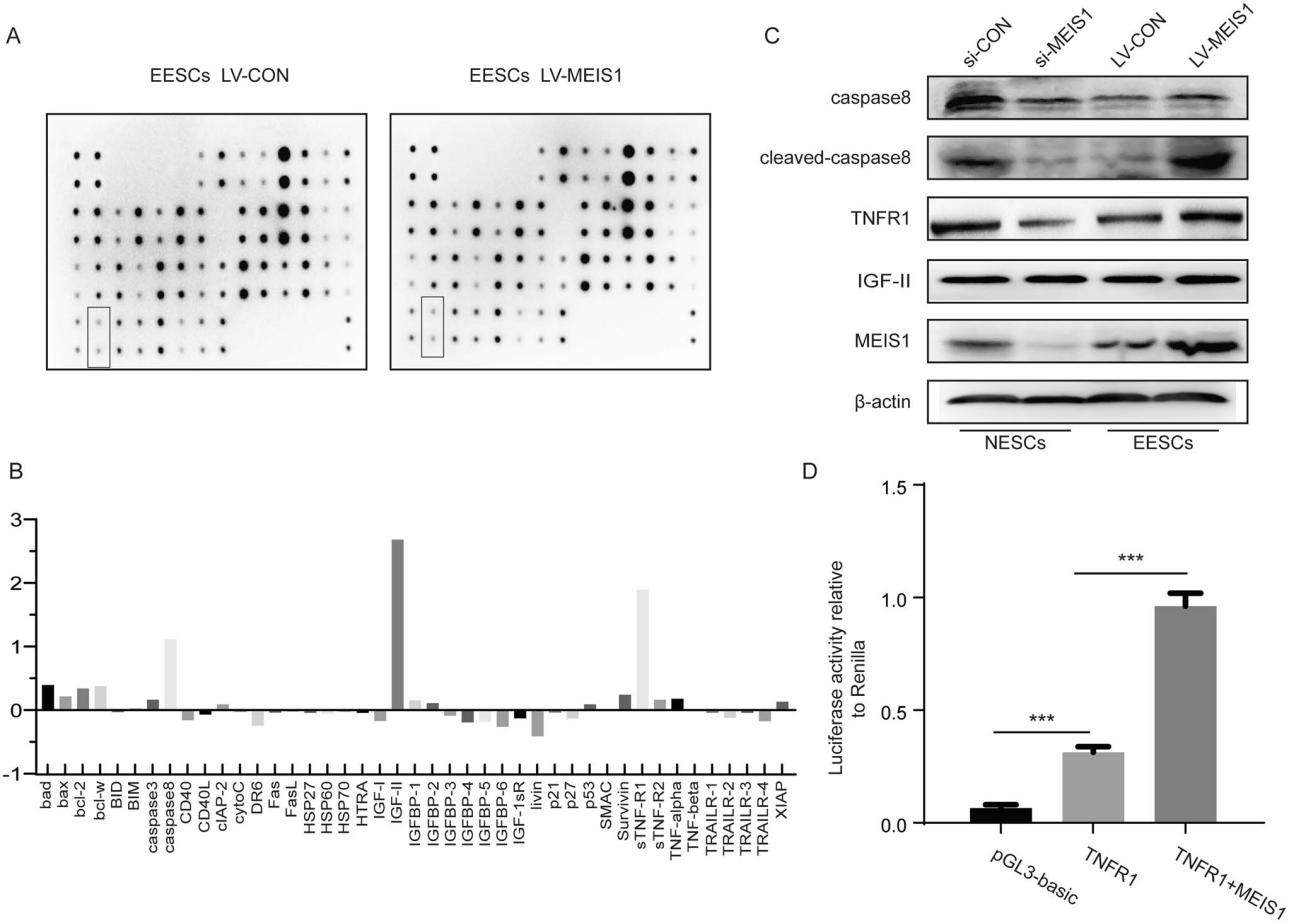
MEIS1 could activate TNFR1-caspase8 apoptosis pathway in ectopic endometrium tissues. (**A**) Total proteins of 1 × 10^7^ EESCs treated by LV-CON or LV-MEIS1 were detected by RayBio Human Apoptosis Antibody Array. (**B**) Quantitative analysis of the gray scale of apoptosis factor proteins in A. (**C**) Western blotting for MEIS1, TNFR1, caspase8, cleaved-caspase8, IGF-II and β-actin in stromal cells after upregulating or downregulating MEIS1. (**D**) Luciferase activity of TNFR1 promoter reporter plasmids using 293T cells with LV-MEIS1 or not. Renilla luciferase expression plasmid was used to normalize the transfection efficiencies. All luciferase experiments were performed three times in duplicate. ***p < 0.001. NESCs: Normal endometrial stromal cells, EESCs: eutopic endometrial stromal cells

Given that MESI1 could induce the apoptosis of endometrial stromal cells, we used the RayBio^®^ Human Apoptosis Antibody Array to determine the possible MEIS1-activated apoptosis pathway. The results revealed positive relations between upregulated MEIS1 and both TNFR1- and IGF-II-induced caspase cascade in EESCs (Fig. 4A and B). To validate this, MEIS1 was upregulated in EESCs and knocked down in NESCs. Western blot revealed that TNFR1 protein level had a consistent relation with MEIS1 in both NESCs and EESCs, not IGF-II (Fig. 4C). In addition, we also observed that caspase 8 was activated following MEIS1 upregulation (Fig. 4C). Given that MEIS1 was a transcription factor, we hypothesized that MEIS1 might regulate TNFR1 gene expression by enhancing its promoter activity. As shown in Fig. 4D, the luciferase activity of TNFR1 promoter plasmid was enhanced by MEIS1, which supported the above hypothesis. Overall, these findings indicated that MEIS1 could induce apoptosis by activating the TNFR1-caspase8 cascade in endometriosis.

### MEIS1 Treatment Inhibits Endometriotic Lesion Progress and had Therapeutic Potential Based on a Mouse Endometriosis Model

**Fig. 5:**
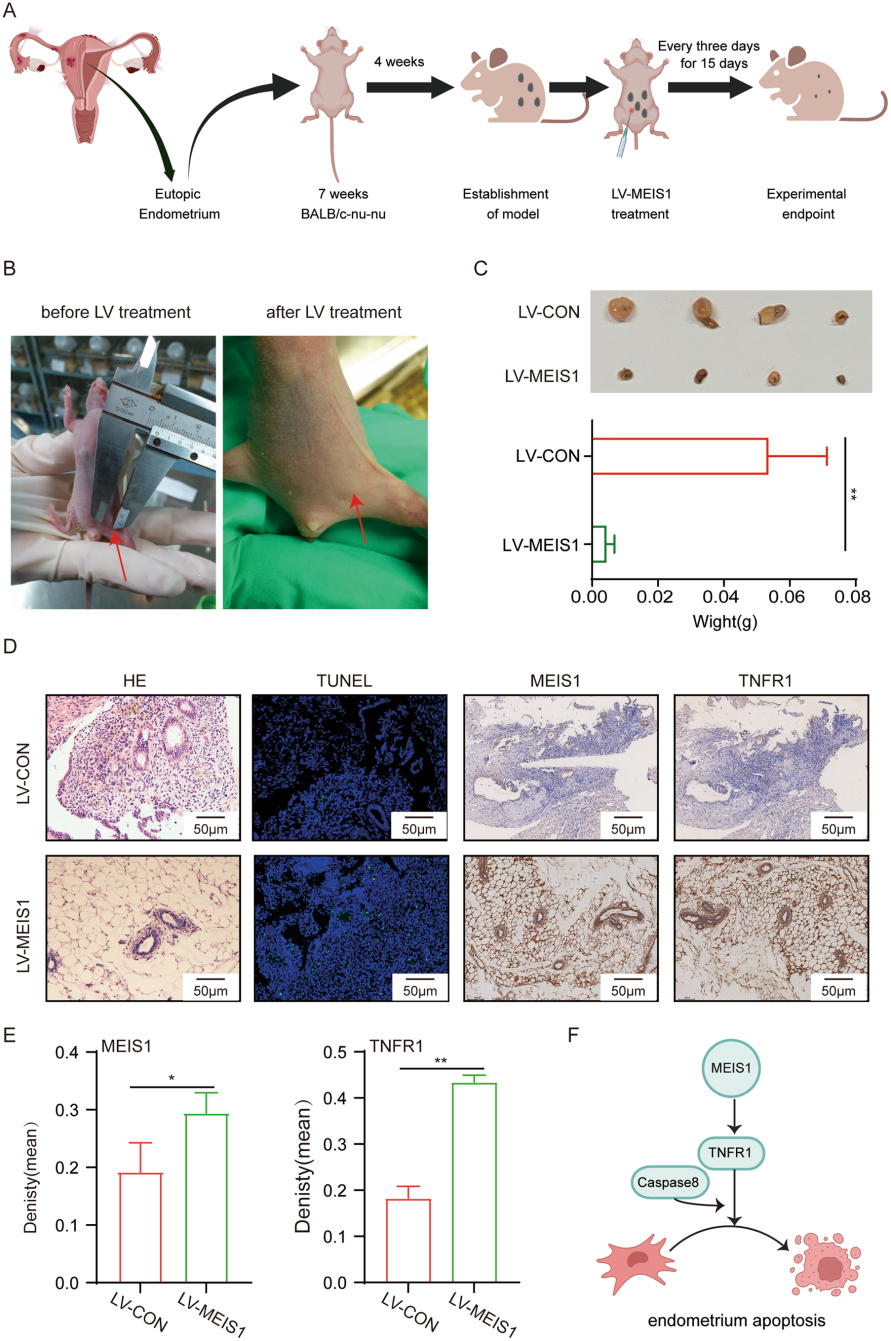
MEIS1 could inhibit endometriotic lesion progress. (**A**) Scheme for the animal study to establish the abdominal endometriosis model in nude mice and to treat of endometriosis lesions by lentivirus. (**B**) The size of endometriosis lesions(marked by red arrow) before and after LV-MEIS1 treatment. (**C**) Endometriosis lesions image and weight at 15 days with LV-MEIS1 and LV-Con treatment. **p < 0.001. (**D**) H&E staining, TUNEL staining, and immunohistochemical detection of MEIS1 and TNFR1 in endometriosis lesions from nude mice. (**E**) The density (mean) of MEISI and TNFR1 proteins immunohistochemistry staining by using Image Pro-Plus (IPP). **p < 0.01 and ***p < 0.001. (**F**) Schematic of mechanisms of MEIS1 enhanced endometriosis cell apoptosis via activating the TNFR1-caspase cascade. H&E: haematoxylin and eosin; TUNEL: terminal deoxynucleotidyl transferase-mediated dUTP-biotin nick end labelling

To identify whether upregulated MEIS1 could inhibit endometriosis growth in vivo, a mouse model was set up by transplanting and suturing eutopic endometrial fragments onto the peritoneum surface. The model was established four weeks after transplantation, then LV-CON or LV-MEIS1 was conducted by intraperitoneal injection every three days for 15 days (Fig. 5A). LV-MEIS1 treatment significantly decreased mouse endometriotic lesion size and weight (Fig. 5B and C). Immunostaining demonstrated that MEIS1 and TNFR1 were significantly increased in MEIS1-treated mouse endometriotic lesion (Fig. 5D and E). Increasing apoptosis was observed in the MEIS1-treated model group by TUNEL staining (Fig. 5D). Importantly, biochemical liver, and renal indicators showed that LV-MEIS1 did not damage primary organ functions (Supplementary Fig. 3). Our data indicated that MEIS1 could reduce the endometriotic lesions by apoptotic signaling pathway activation.

## Discussion

Under physiological conditions, apoptosis can maintain endometrial homeostasis by eliminating the functional layer of senescent cells before formation of necrotic tissue [27], which also prevents the migration and stacking of ectopic and eutopic endometrial cells [7]. However, the apoptotic rate of eutopic endometrial cells is significantly decreased in women with endometriosis [28, 29]. Despite a burgeoning literature indicating that apoptosis plays a pivotal role in the development and progression of endometriosis, its pathogenesis needed further investigation. Herein, we identified MEIS1 as an apoptosis-related gene in endometriosis by analyzing our previously published data, RNA-seq and public endometriosis database (GES7305). Overexpression of MEIS1 promoted apoptosis of endometrial stromal cells in endometriosis by activating the TNFR1-caspase8 pathway (Fig. 5F). Furthermore, we also demonstrated that improved MEIS1 could shrink mouse endometriotic lesions.

Our pooling-based GWAS [13], combined with RNA sequencing and a published endometriosis database (GSE7305) [14], revealed an OMA-related gene—MEIS1. It plays a crucial role in transcriptional regulation of various functional genes. Herein, MEIS1 expression was significantly decreased in eutopic and ectopic endometrial tissues of different phases using quantitative real-time PCR, which is consistent with results from Hu et al. [26], and MEIS1 protein level in eutopic endometrial tissues was also decreased, as it was in OMA tissues. IHC revealed that nuclear and cytoplasmic MEIS1 expression in stromal and glandular cells of endometrial tissues without endometriosis, which are basically the same as Dintilhac’s [30]. However, in eutopic and ectopic tissues, there is negative staining of MEIS1 in nucleus. Different subcellular localization of MEIS1 play diversity roles in cellular functions and biological processes. MEIS1 in the nucleus, not in cytoplasm, promoted proliferation of hair matrix cell [31]. Our results imply that the loss of MEIS1 in the nucleus may contribute to pathogenesis of endometriosis.

LV-MEIS1 treatment significantly increased MEIS1 expression. Upregulation of MEIS1 expression in eutopic stromal cells promoted cell death, consistent with a murine model of endometriosis. Meanwhile, siRNA of MESI1 was used to decrease MEIS1 expression. Downregulation of MEIS1 expression in normal stromal cells induced cell proliferation. Screening by apoptotic chips, we identified that MEIS1 could upregulate protein expression of TNFR1, which might be achieved by enhancing TNFR1 promoter activity. Ultimately, MEIS1 initiated the caspase pathway, leading to apoptosis. This is similar to the finding by Wermuth et al., that MEIS1 could mediate caspase-dependent apoptosis, which is repressed by co-expression of HoxA9 in rodent and human cell lines [20]. Tamai et al. also confirmed that MEIS1 activation could initiate the caspase 8–caspase 3 apoptotic pathway in mixed-lineage leukemia/AF4-positive acute lymphoblastic leukemia [32]. MEIS1 protein could also suppress proliferation, migration, invasion, and metastasis of other tumors [23, 33, 34]. To our knowledge, ours is the first study to explore the relations between MEIS1 and TNFR1 in endometriosis.

Our observations established that a mouse model of endometriosis could identify whether MEIS1 treatment had therapeutic effects on endometriosis. Our in vivo study showed that MEIS1 overexpression by intraperitoneal administration could reduce endometriotic lesion size in this model. Furthermore, body weight, liver functions and kidney functions of treatment group mice did not differ significantly from control mice, bolstering confidence in the integrity of this model. In short, promoting MEIS1 function may decrease endometriosis progress.

This study was not without shortcomings. The primary stromal cell culture may have had some altered gene expressions, which were not evaluated herein. To reduce this impact, we tried to complete gene function detection within one week of in vitro culture.

In conclusion, this study revealed the function of MEIS1 in the pathogenesis of endometriosis, based on its identification in previous genomic studies. To our knowledge, this study is the first to explore downregulated MEIS1 in tissues from patients with endometriosis, revealing that it may inhibit the TNFR1-mediated apoptotic pathway of the caspase family, promoting ectopic endometrial cell growth and lesion formation. Targeting MEIS1 may become a future nonhormonal endometriosis therapy alternative.

## Electronic Supplementary Material

Below is the link to the electronic supplementary material.

Supplementary Material 1

## Data Availability

The data used in this study are available from the corresponding author upon reasonable request.
