# Supplementary material for: MEIS1-mediated Apoptosis via TNFR1 in Endometriosis
Source: Reprod Sci. 2025 Feb 11;32(3):716–27. doi: 10.1007/s43032-025-01801-1 (PMC11870962; doi:10.1007/s43032-025-01801-1)
Supplement: Supplementary file 1 — Supplementary Material 1 [file 43032_2025_1801_MOESM1_ESM.pdf]

Supplementary Fig. 1

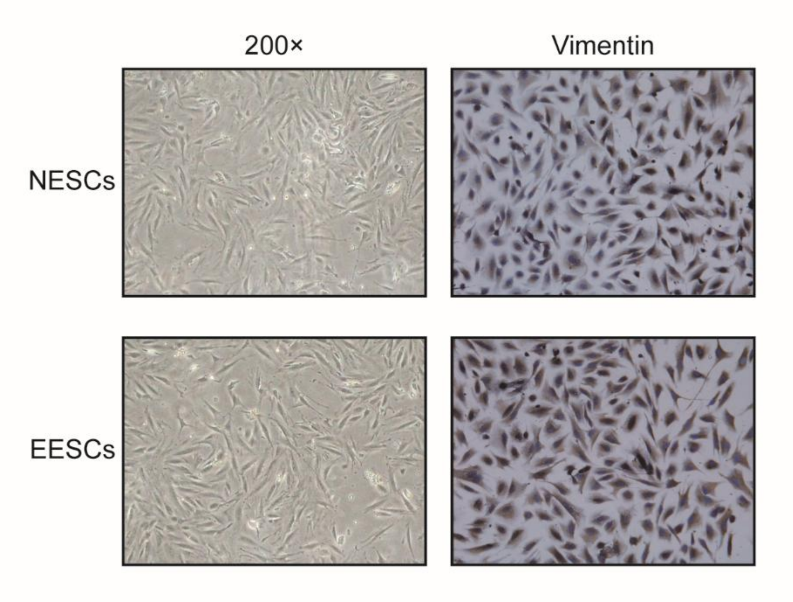

Supplementary Fig. 1: Normal endometrial stromal cells and eutopic endometrial stromal cells were identified by immunocytochemistry staining for Vimentin. Scale bars:

NESCs: Normal endometrial stromal cells, EESCs: eutopic endometrial stromal cells

Supplementary Fig. 2: Heat maps of RNA sequencing from 5 normal endometrium tissues and 5 ovarian endometrioma tissues. OMA: ovarian endometrioma, Nor-E: normal endometrium.

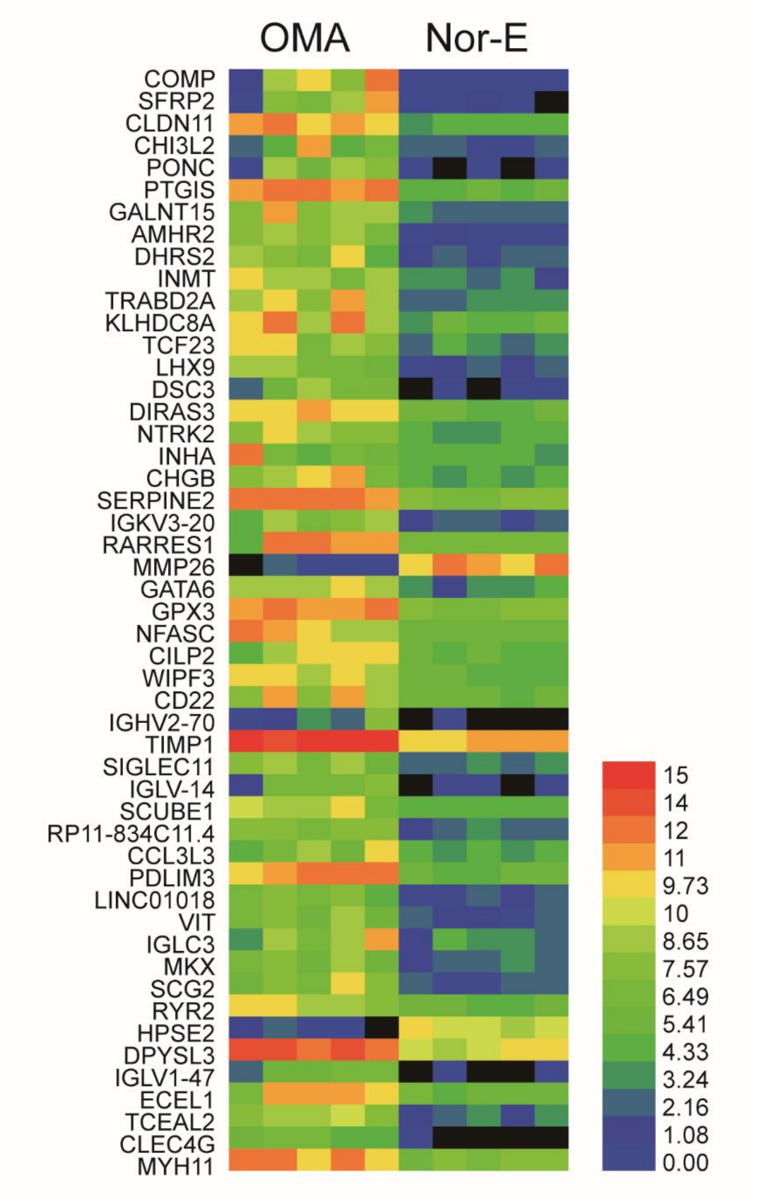

Supplementary Fig. 3

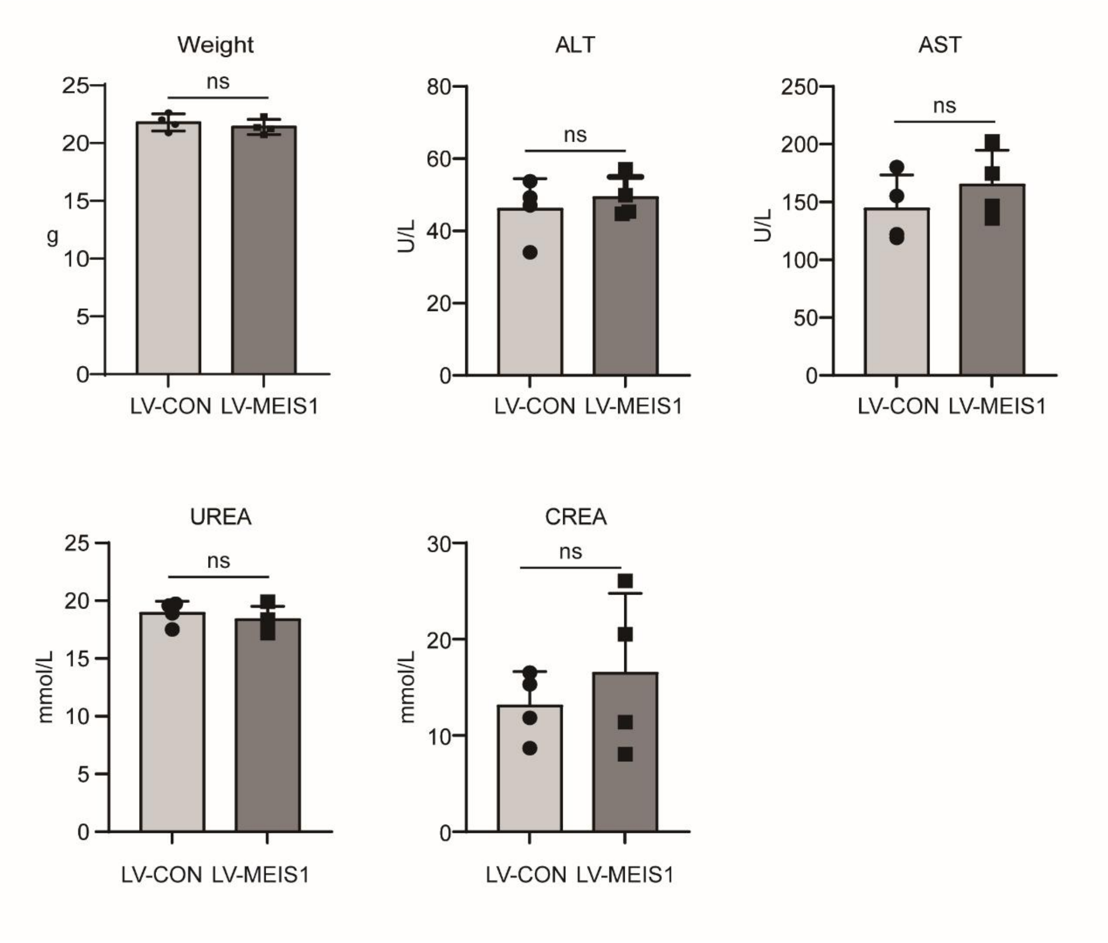

Supplementary Fig. 3: The weight ang the levels of serum ALT (glutamic pyruvic transaminase), AST (glutamic oxalacetic transaminase), UREA and CREA (creatinine) in the two groups of animal study.
